# Supplementary material for: Why do nanowires grow with their c-axis vertically-aligned in the absence of epitaxy?
Source: Sci Rep. 2020 Apr 16;10:6554. doi: 10.1038/s41598-020-63500-y (PMC7162979; doi:10.1038/s41598-020-63500-y)
Supplement: Supplementary file 1 — Supplementary information. [file 41598_2020_63500_MOESM1_ESM.pdf]

Supplementary Information

Why do nanowires grow with their c-axis vertically-aligned in the absence of epitaxy?

Almog R. Azulay,[1] Yury Turkulets,[1] Davide Del Gaudio,[2] R.S. Goldman,[2] & Ilan Shalish[1]*

This file contains the following additional data to support the assertions made in the main text of the paper: a histogram of angle-to-substrate of the nanowires in Fig. 1c, x-ray diffraction of the polycrystalline ZnO layer shown in Fig. 1d, contact potential measured using a Kelvin probe on the oxidized Si wafers before and after thermal oxidation or PECVD oxide growth, an illustration and explanation of the observed growth orientation vs. the substrate charge, and additional TEM/CBED results.

[1]School of Electrical and Computer Engineering, Ben-Gurion University, Beer Sheva 8410501, Israel. [2]Department of Materials Science and Engineering, University of Michigan, 2300 Hayward St., Ann Arbor 48109 MI USA.
*email: shalish@bgu.ac.il

Nanowire Angle

Angles of 107 wires from Fig. 1c were measured using the software ImageJ.[1] The observed angles were 90.95±4.65 degrees. Fig S1 shows a bar graph of the frequencies with 5-degree binning. Figure S2 shows cress-sectional SEM view of ZnO nanowires gown on thermal oxide (panel A) and on PECVD oxide (panel B). The top insert in each panel shows a top view, and the bottom insert shows the corresponding histogram.

X-ray Diffraction for Fig. 1d

Figure S2 shows two-theta x-ray diffraction obtained from the sample of Fig. 1(d) in the paper. This ZnO layer was grown on a thermally oxidized Si(100) with oxide thickness of about 2 µm. The diffraction shows a single peak at 34.38° which fits the (0002) diffraction in R050492 powder diffraction file.[2] This shows that albeit the polycrystalline appearance, the grains are c-oriented.

Oxide Charge

In this work, Si wafers were thermally oxidized to produce a positively-charged oxide and PECVD-oxidized to produce negatively charged oxide. To verify the oxide charge, we conducted multiple measurements of the contact potential on 10 samples before oxidation, 10 samples after thermal oxide growth, and 10 samples after PECVD oxide growth. The Kelvin probe is brought to a distance of 0.3 mm from the sample surface forming a capacitor wherein one plate is the sample and the other plate is the probe. The probe is vibrated at a constant frequency and this varies sinusoidally the clearance between the plates (d). This variation of d produces an alternating (AC) current in the capacitor. The voltage on the probe is then changed until it matches the surface voltage on the sample, at which point the charge on the capacitor is zero and the AC current nullifies. This way the surface voltage (also known as the "contact potential") of the sample is measured. If a charged oxide layer is added within this capacitor, it will make the surface voltage more positive relative to its original value in the case of positively charged oxide, and more negative in the case of negatively charged oxide.

Figure S3 shows a statistical box plot for each of the statistical samples relative to the mean value measured before oxidation. The figure shows that thermal oxidation makes the contact potential more positive, while PECVD oxide makes it more negative. This confirms qualitatively the presence of positive charges in the thermal oxide and negative charges in the PECVD oxide, as expected. This result confirms in our samples what has been known for many decades since the early days of the transistor.[3,4,5,6] The theory underlying the Kelvin probe is described in great detail in a review by Kronik and Shapira.[7] Additional review of various oxide charge measurement methods using a Kelvin probe are described in a textbook by Schroder.[8]

Growth Orientation vs. Substrate Charge

In Fig. S4, we show a "ball and stick" structure of a ZnO unit cell. The Zn-terminated polar face is in the (0002) direction and is hosts negative polar charge, while the O-terminated face points in the (000-2) direction and hosts a positive polar charge. A polar crystal may be able to stabilize its positively charged face by placing it face-down on a negatively charged substrate. This explains why on PECVD-grown oxide (negatively charged), we observe the growth to be oriented in the (0002) direction (Zn-terminated face is up).

Determining the sign of polar charge on each of the polar faces

Polar surface charge in wurtzite materials is not simply defined by the charge of the ions at its surfaces, but rather is defined by the arrangement of the ions in the lattice. Lattice asymmetry in C-direction of typical wurtzite materials (e.g., ZnO, GaN), induces a permanent dipole moment. As a result of this asymmetrical ion arrangement, the Zn-face in ZnO becomes the negative pole of the dipole, holding the negative polar charge, and the O-face becomes the positive pole, holding the positive polar charge (this is despite of the fact that Zn is the cation and O is the anion). The same is true for GaN.[9]

The method to calculate the spontaneous polarization is described, for example, by Nann *et* Schneider.[10] The polarity vector is defined as pointing in the direction of the positive polar charge. To find out the actual direction of the polarity vector, we first define a standard unit vector, $\hat{p}$, pointing at the cation-face (0002) direction, as our reference. Next, we sum up the *projections* of the dipole moments of the various chemical bonds on this unit polarity vector. In the wurtzite crystal structure, each cation is connected to four anions (and also each anion is connected to 4 cations) in a tetrahedral structure (see Fig. s5). One of the four chemical bonds in the tetrahedral is parallel to the polar axis (having an angle of 0° or 180° to the nominal polarity vector), while the three other

bonds are at a certain, material-specific, angle, θ, to the unit polarity vector. To calculate the polarity, P, one has to sum up the projections of the dipole moments, induced by the four chemical bonds, on the polar axis and divide the sum by the volume, v, of the cell:

$$P = \frac{1}{v}\sum_{i=1}^{4} q_i l_i \cos{(\theta_i)}$$

where $q_i$and l_i are the dipolar charge, and the length, of the i-th bond, and θ_i is the angle of the i-th bond relative to the nominal polar vector. In the case of ZnO, if we use the numbers given by Nann et Schneider (adopting the angles to the tetrahedron shown in Fig. s5), then $q_1 = q_2 = q_3 = q_4$ $l_1 = 0.1988\ nm$, $l_2 = l_3 = l_4 = 0.19745\ nm$, $\theta_1 = 180°$, and $\theta_2 = \theta_3 = \theta_4 = 71.86°$. Using these numbers, we get

$$P = \frac{q_1}{v}[0.1988 \cdot (-1) + 3 \cdot 0.19745 \cdot 0.31134] = -\frac{q_1}{v} \cdot 0.0143778 < 0$$

Since the calculated polarity comes out negative, this means that the actual polarity vector is pointing opposite to our reference unit polar vector, i.e., the positive polar charge is actually on the oxygen face. Projections on other axes are symmetric and cancel each other. We note that the numbers, and the model, taken from the above reference are approximated for the simple case of point charges. More rigorous calculations yield more accurate numbers, and several such calculations have been reported.[11,12,13,14,15,16,17,18,19,20] Regardless of the accuracy, the sign of the spontaneous polarization in ZnO is always negative, and this means that the Zn-face carries the negative polar charge.

Understanding of this concept is of critical importance in electronic devices of polar materials. By far, the most common polar semiconductor device material today is GaN. In GaN, the negative polar charge on the Ga-face is a key for the operation of the GaN high electron mobility transistor (HEMT). In the common AlGaN/GaN structure, the layers are grown in +C (0002) direction. The polar electric field thus created gives rise to a 2-dimetional electron gas at the AlGaN\GaN interface. If, instead, the growth is carried out in the -C $(000\bar{2})$ direction, the resulting polar electric field that gives rise to a 2-dimentional hole gas.[21,22]

Additional TEM/CBED results

In Fig. S6, we add additional CBED data. All the data on each of the oxides, both here and in the paper, were obtained from the same growth run. In thin wires the CBED pattern is blurred, and this makes it more difficult to compare it to simulation (see e.g. the thermal oxide CBED in line 4 below). For this reason, most of the CBED were acquired from thick wires. For each wire, CBED was acquired from both the bottom and the top of the wire to make sure that the polarity is identical throughout the wire. However, for brevity, we show only a single pattern. In each figure, panel A shows the top end of the wire, panel B shows the bottom end, panel C shows the SAED pattern, and panel D compares the CBED pattern with a simulated CBED pattern.

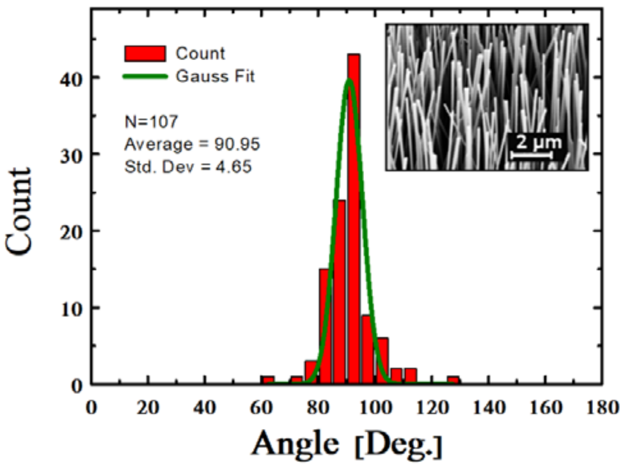

Figure s1. Histogram of angles of the nanowires in Fig. 1(c) in the paper. Angles were measured using the ImageJ software on N=107 nanowires. The average angle was 90.95 ± 4.65 °.

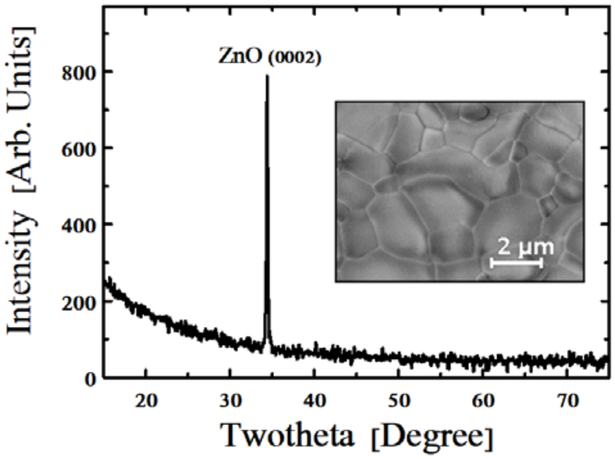

Figure s3. Two-theta x-ray diffraction from the Si(100)\SiO2\ZnO sample of Fig. 1(d) (SEM image of the surface is shown in the inset). The diffraction shows a single peak that fits the position of ZnO(0002) in R050492 powder diffraction file.

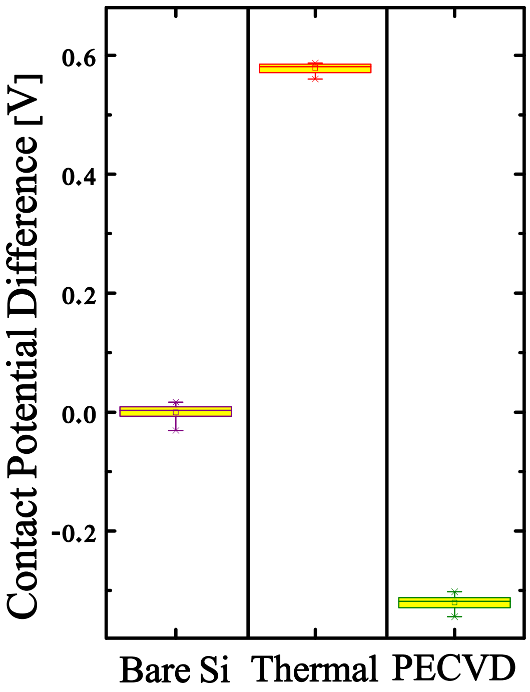

Figure s4. Box plots of contact potential measured using a Kelvin probe on the oxidized Si wafers before ("Bare Si") and after thermal oxidation ("Thermal") or PECVD oxide growth ("PECVD"). The average value of contact potential before oxidation is set as zero for comparison.

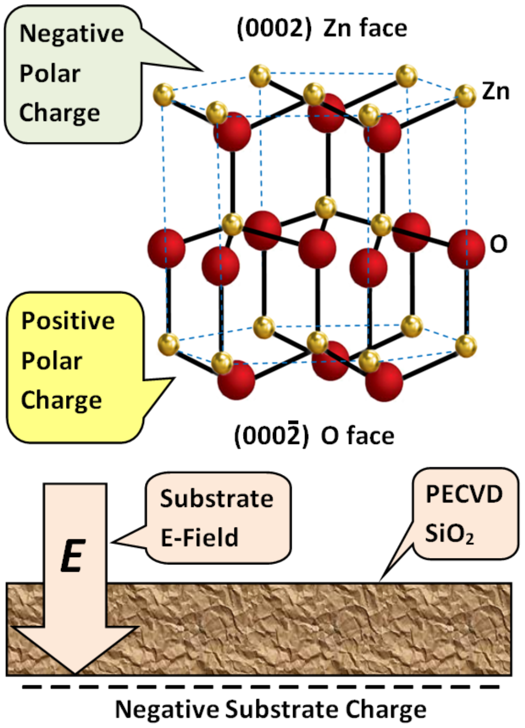

Figure s5. Ball and stick model of the ZnO unit cell showing the polar crystal faces, their orientation, their polar charges, and the resulting growth orientation when grown of a negatively charged substrate.

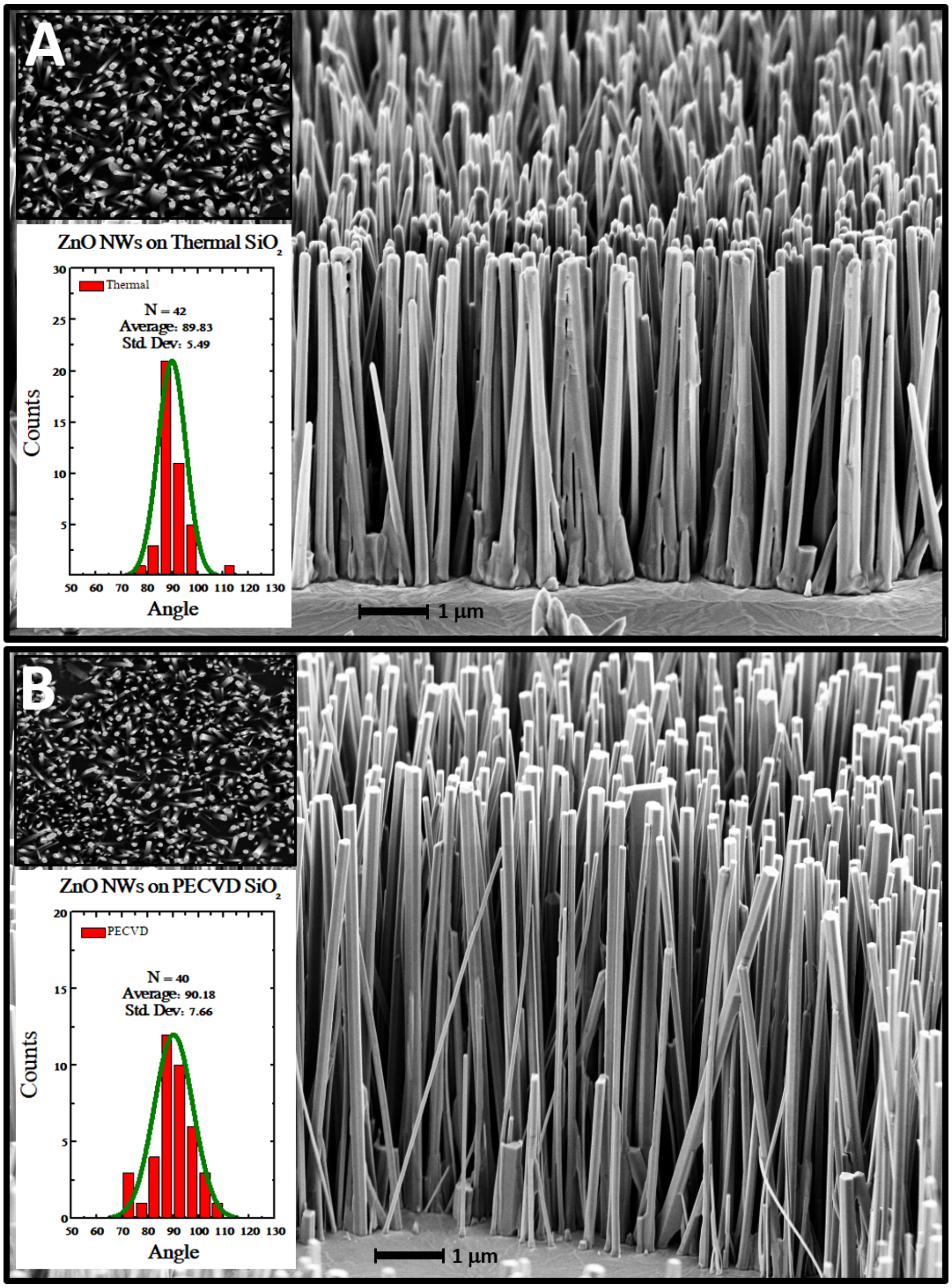

Figure s2. cress-sectional SEM view of ZnO nanowires gown on thermal oxide (panel A) and on PECVD oxide (panel B). The top insert in each panel shows a top view, and the bottom insert shows the corresponding angle histogram.

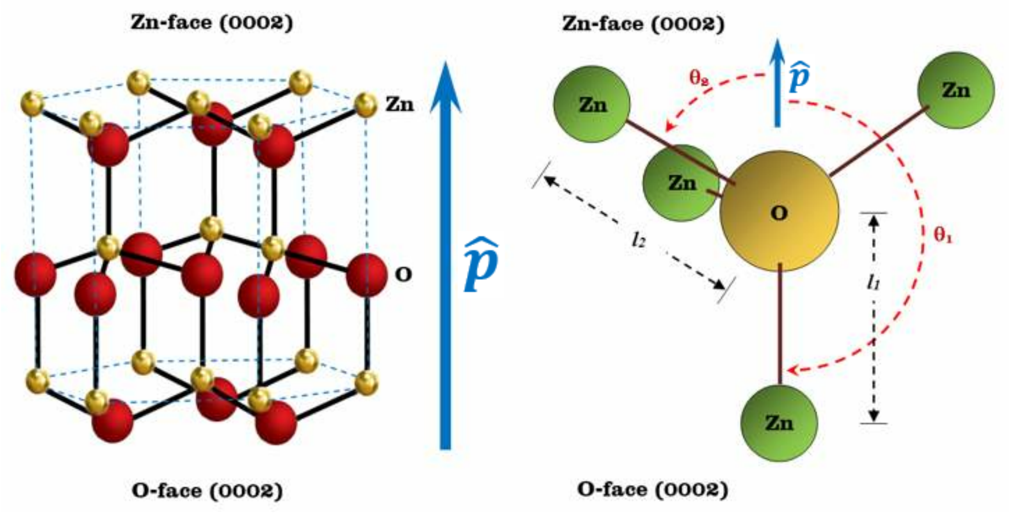

Figure s6. Ball and stick cartoons: [a] ZnO crystal structure (left), and [b] arrangement of dipole in ZnO tetrahedral

Figure s7. Additional TEM/CBED results obtained from the same growth run as the results shown in Fig. 2 in the paper

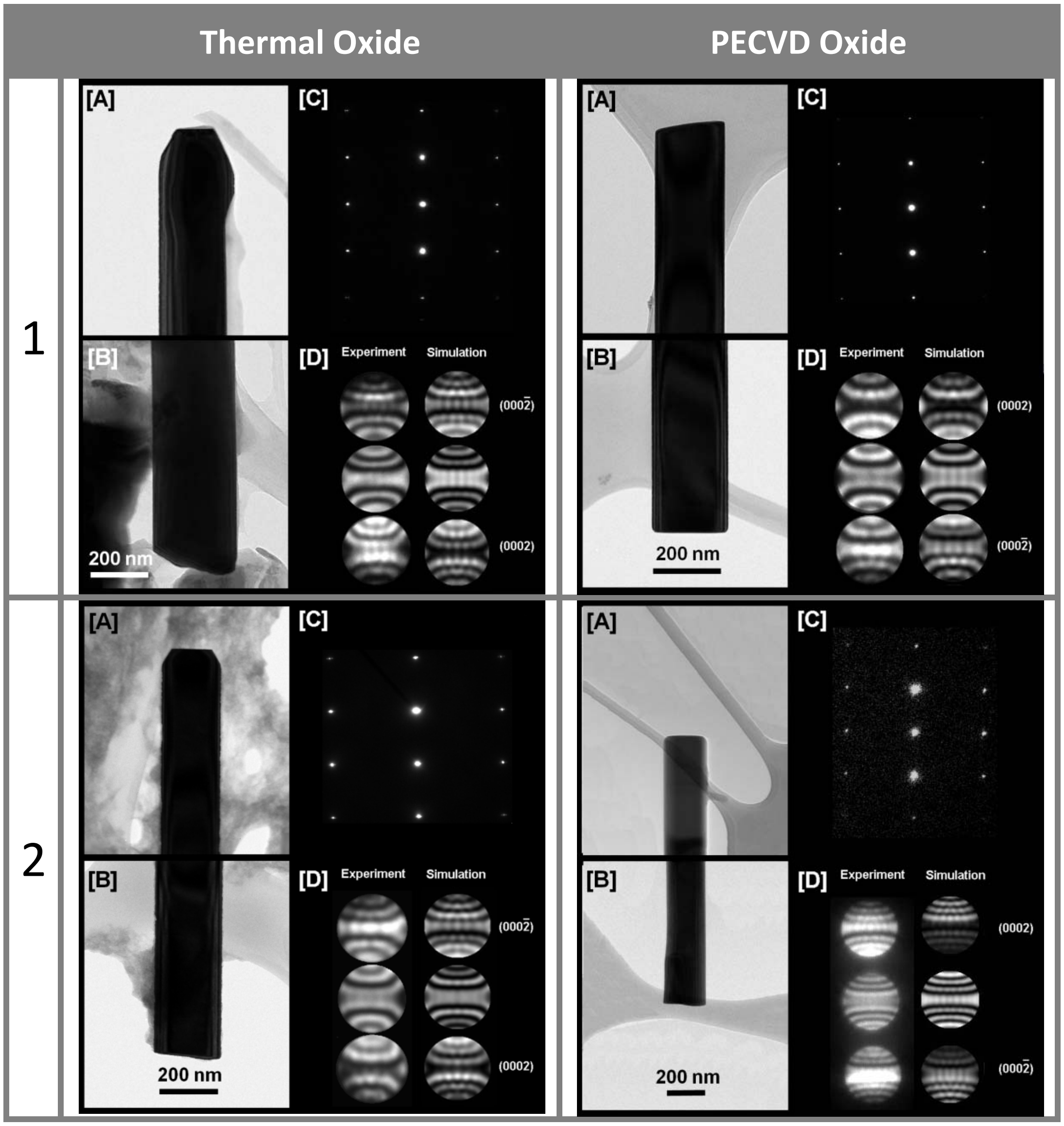

	Thermal Oxide	PECVD Oxide
3	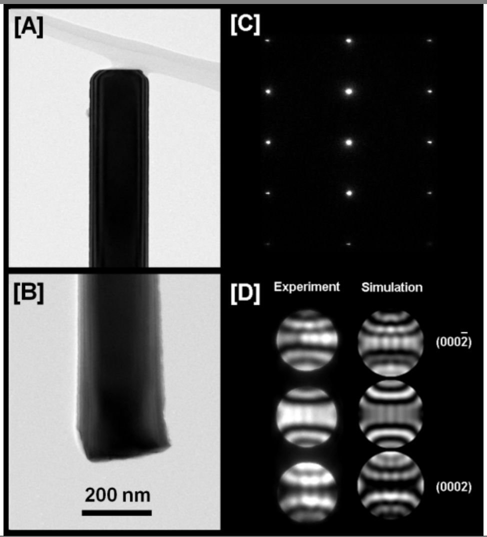	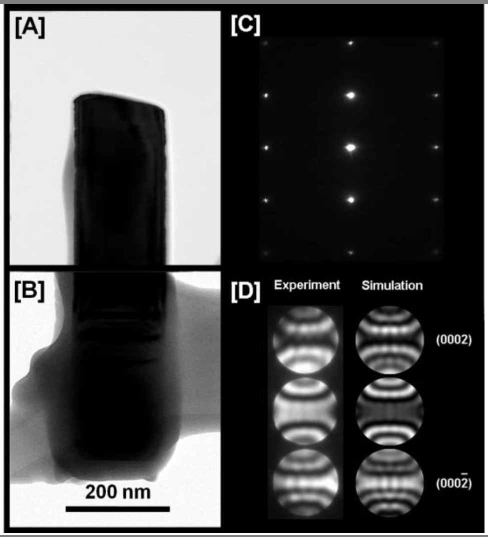
4	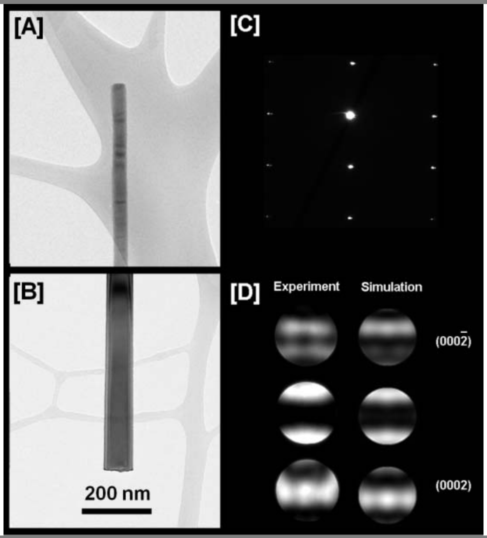	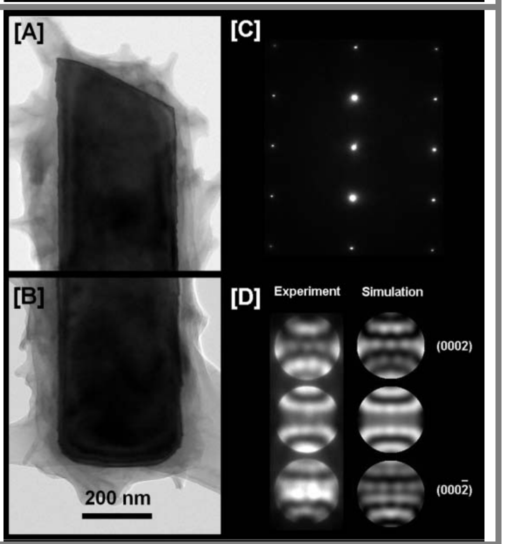
5	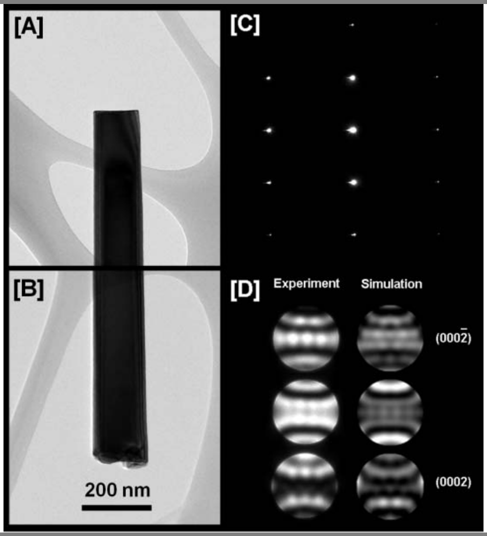	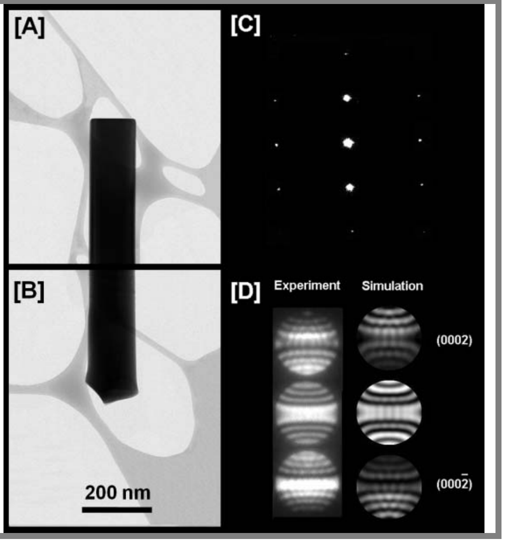

References

1. ImageJ is a public domain Java image processing program provided free of charge by the National Institute of Health (NIH). It may be downloaded at https://imagej.nih.gov/ij/
2. RRUFF Project database. http://rruff.info/zincite/R050492
3. M.M. Atalla, E. Tannenbaum, E.J. Scheibner. Stabilization of Silicon surfaces by thermally grown oxides. *Bell Sys. Tech. J*. **38**, 749 (1959). DOI: 10.1002%2Fj.1538-7305.1959.tb03907.x
4. B.E. Deal, M. Sklar, A.S. Grove, E.H. Snow. Characteristics of the Surface-State Charge of Thermally Oxidized Silicon. *J. Electrochem. Soc*. **114**, 266 (1967). DOI: 10.1149/1.2426565
5. B.E. Deal. The Current Understanding of Charges in the Thermally Oxidized Silicon Structure. *J. Electrochem. Soc.* **121**, 198C (1974). DOI: 10.1149/1.2402380
6. A. Boogaard , A.Y. Kovalgin, R.A.M. Wolters. Net negative charge in low-temperature SiO2 gate dielectric layers. *Microelectron. Eng.,* **86**, 1707 (2009). DOI: 10.1016/j.mee.2009.03.124
7. L. Kronik, Y. Shapira. Surface photovoltage phenomena: theory, experiment, and applications. *Surf. Sci. Rep.* **37**, 1 (1999). DOI: 10.1016/S0167-5729(99)00002-3
8. D.K. Schroder. 2006. *Semiconductor Material and Device Characterization*, 3rd ed. New Jersey: Wiley. ISBN: 978-0-471-73906-7. p. 538.
9. U. K. Mishra, J. Singh, *Semiconductor Device Physics and Design*, (Springer, Dordrecht, Netherlands, 2008), p. 388.
10. T. Nann, J. Schneider. Origin of permanent electric dipole moments in wurtzite nanocrystals. *Chem. Phys. Lett.* **384**, 150 (2004).
11. F. Bernardini, V. Fiorentini, D. Vanderbilt. Spontaneous polarization and piezoelectric constants of III-V nitrides. *Phys. Rev. B* **56**, R10024 (1997).
12. A. Dal Corso, M. Posternak, R. Resta, A. Baldereschi. Ab initio study of piezoelectricity and spontaneous polarization in ZnO. *Phys. Rev. B* **50**, 10715 (1994).
13. K. Shimada, T. Sota, K. Suzuki. First-principles study on electronic and elastic properties of BN, AlN, and GaN. *J. Appl. Phys.* **84**, 4951 (1998).
14. A. Zoroddu, F. Bernardini, P. Ruggerone, .V. Fiorentini. First-principles prediction of structure, energetics, formation enthalpy, elastic constants, polarization, and piezoelectric constants of AlN, GaN, and InN: Comparison of local and gradient-corrected density-functional theory. *Phys. Rev. B* **64**, 045208 (2001).
15. M.Catti,Y.Noel, R.Dovesi. Full piezoelectric tensors of wurtzite and zinc blende ZnO and ZnS by first-principles calculations. *J. Phys. Chem. Solids* **64**, 2183 (2003).
16. S. Liu, R.E. Cohen. Origin of Negative Longitudinal Piezoelectric Effect. *Phys. Rev. Lett*. **119**, 207601 (2017).
17. A. Schleife, F. Fuchs, J. Furthmüller, F. Bechstedt. First-principles study of ground- and excited-state properties of MgO, ZnO, and CdO polymorphs. *Phys. Rev. B* **73**, 245212 (2006).
18. P. Lawaetz. Stability of the Wurtzite Structure. *Phys. Rev. B* **5**, 4039 (1972).
19. A. Seko, F. Oba, A. Kuwabara, I. Tanaka. Pressure-induced phase transition in ZnO and ZnO−MgO pseudobinary system: A first-principles lattice dynamics study. *Phys. Rev. B* **72**, 024107 (2005).
20. X.F. Fan, H.D. Sun, Z.X. Shen, J.-L. Kuo, Y.M. Lu. A first-principle analysis on the phase stabilities, chemical bonds and band gaps of wurtzite structure AxZn1−xO alloys (A = Ca, Cd, Mg). *J. Phys.:Condens. Matter* **20**, 235221 (2008).
21. U.K. Mishra, L. Shen, T.E. Kazior, Y.F. Wu. GaN-Based RF power devices and amplifiers. *Proc. IEEE* **96**, 287 (2008).
22. O. Ambacher and V. Cimalla, “Polarization induced effects in GaN-basedheterostructures and novel sensors” in *Polarization Effects in Semiconductors*, edited by C. Wood and D. Jena (Springer, New York,2007), pp. 27–110.
